# Quantification of muscle tone by using shear wave velocity during an anaesthetic induction: a prospective observational study

**DOI:** 10.1186/s12871-023-02358-9

**Published:** 2023-11-29

**Authors:** Hidehisa Saito, Shigekazu Sugino, Shoichiro Moteki, Akihiro Kanaya, Masanori Yamauchi

**Affiliations:** Department of Anesthesiology and Perioperative Medicine, Tohoku University Graduate School of Medicine, 2-1, Seiryo-Machi, Aoba-Ku, Sendai, Miyagi 980-8575 Japan

**Keywords:** Muscle tonus, Elasticity imaging techniques, Muscle relaxation, Neuromuscular blockade, General anesthesia

## Abstract

**Objectives:**

The quantitative assessment of muscle stiffness or weakness is essential for medical care. Shear wave elastography is non-invasive ultrasound method and provides quantitative information on the elasticity of soft tissue. However, the universal velocity scale for quantification has not been developed. The aim of the study is to determine the shear wave velocities of abdominal muscle during anesthetic induction and to identify methods to cancel the effects of confounders for future development in the quantitative assessment of muscle tone using the universal scale.

**Methods:**

We enrolled 75 adult patients undergoing elective surgery with ASA-PS I – III in the period between December 2018 and March 2021. We measured and calculated the shear wave velocity (SWV) before and after opioid administration (i.e., the baseline at rest and opioid-induced rigidity condition), and after muscle relaxant administration (i.e., zero reference condition). The SWV value was adjusted for the subcutaneous fat thickness by our proposed corrections. The SWVs after the adjustment were compared among the values in baseline, rigidity, and relaxation using one-way repeated-measures ANOVA and post hoc Tukey–Kramer test. A *p*-value of < 0.05 was considered to be statistically significant. UMIN Clinical Trials Registry identifier UMIN000034692, registered on October 30, 2018.

**Results:**

The SWVs in the baseline, opioid-induced rigidity, and muscle relaxation conditions after the adjustment were 2.08 ± 0.48, 2.41 ± 0.60, and 1.79 ± 0.30 m/s, respectively (*p* < 0.001 at all comparisons).

**Conclusion:**

The present study suggested that the SWV as reference was 1.79 m/s and that the SWVs at rest and opioid-induced rigidity were ~ 10% and ~ 30% increase from the reference, respectively. The SWV adjusted for the subcutaneous fat thickness may be scale points for the assessment of muscle tone.

## Introduction

Assessing muscle stiffness is essential for medical care. Over-exercise or poor posture causes chronic muscle tightness and affects daily activities and quality of life [1]. In orthopedic and rehabilitation medicine, sprains and strains are the most common reasons for muscle stiffness [2]. Pain, anxiety, and depression cause increased muscle tension, leading to significant myofascial pain [3]. Meanwhile, muscle weakness is also observed in medical care. Neuromuscular disorders, hypothyroidism, or electrolyte imbalances produce muscle weakness [4–6]. In intensive care, ICU-acquired weakness after mechanical ventilation in the long duration is the strong risk factor for death [7]. Physicians need the quantitative determination of muscle stiffness or weakness for patients.

Shear wave elastography is a non-invasive ultrasound method and provides quantitative information on the elasticity of soft tissue [8]. This method uses an acoustic pulse to produce shear waves, propagating perpendicularly to the muscle. Previous research reported the linear relationship between shear wave velocity and muscle stiffness [9]. The faster velocity reflects the increased muscle tone [10]. However, the universal velocity scale for quantitative assessment by healthcare providers has not been developed.

In the current study, we prepared two situations: iatrogenic muscle rigidity and complete muscle relaxation. The iatrogenic rigidity was produced by the administration of opioids. It is called opioid-induced lead-pipe rigidity during an anesthetic induction for surgery [11]. Also, completely relaxing muscle is produced by the administration of muscle relaxant during an anesthetic induction. The aim of the study is to determine the shear wave velocities in these situations and to identify methods to cancel the effects of confounders for the future development in the quantitative assessment of muscle tone by the universal scale. This article was prepared following the STROBE statement [12].

## Materials and methods

### Clinical trial registration

This single-center clinical trial was approved by the Institutional Review Board of Tohoku University Hospital (approval no. 2018–1-566, October 31, 2018) and registered prior to patient enrolment in the UMIN Clinical Trial Registry (UMIN-CTR, identifier: UMIN000034692, October 30, 2018).

### Participants

We conducted a prospective cohort study. We screened patients for study eligibility if they were adult (> 20 years of age) patients with ASA physical status classification of I – III, who were scheduled for elective surgery under general anesthesia at the Tohoku University Hospital from December 2018 to March 2021. Patients with neuromuscular disease or allergy to neuromuscular relaxant were excluded. Written informed consent was acquired from all patients before study participation. Information regarding pre-existing medical history, age, sex, height, weight, ASA physical status classification, and type of surgery, was collected from electronic medical record system.

### Experimental protocol in induction of general anesthesia

All patients were in the supine position on an operating table. We added four treatments every 5 min for general anesthesia induction. First, 6 L/min of oxygen was administered to the patient via a face-mask. Second, at 5 min after the start of preoxygenation, 0.5 mg/kg/min of remifentanil was administered intravenously. Third, at 5 min after the start of remifentanil administration, general anesthesia was induced by 0.5 – 2 mg/kg of propofol, followed by 3—6 mg/kg/hrs continuous infusion, or 1–2% sevoflurane, or 3% desflurane. Muscle relaxant (0.8 mg/kg of rocuronium bromide) was administered intravenously to abolish muscle tone. Finally, at 5 min after anesthetic induction, the airway was secured by endotracheal intubation, and positive mechanical ventilation was performed in all patients. Muscle tone was evaluated at three time points: 1) oxygen alone before remifentanil administration (baseline), 2) remifentanil administration before anesthetic induction (opioid-induced rigidity), 3) after anesthetic induction (complete muscle relaxation). The primary endpoint was muscle tone in the complete muscle relaxation. The secondary endpoint was the tone in the opioid-induced rigidity.

### Ultrasound examination for evaluation in muscle tone

A diagnostic ultrasound system (Acuson S3000, Siemens Medical Solutions USA, Inc., Malvern, PA, USA) with a linear array transducer (9L4, 4 – 9 MHz, Siemens) was used to evaluate the muscle tone. An examiner scanned the abdominal rectus muscle near the umbilicus, and obtained B-mode images at the horizontal axis. Next, the examiner used Virtual Touch Imaging mode to visualize the muscle tone of the abdominal rectus muscle. And then, the examiner applied Virtual Touch Imaging Quantification mode for shear wave elastography. Three regions of interest were determined in the image. Velocity value, which reflects the muscle elasticity, was measured in each region by using shear wave elastography. At the longitudinal axis, the three values of the velocities were measured again. Average of the velocity values in six regions was used to calculate the shear wave velocity (SWV). All measurements were performed by a single examiner, a board-certified anaesthesiologist, who practiced on 13 volunteers before the study commenced. The ultrasound image was saved as in JPEG format to prepare for later verification. In this digital image, subcutaneous fat thickness was measured in the same six areas was in the image in which the velocity values were measured. We used the mean of six values of thicknesses in subsequent analysis.

### Statistical analysis

Numerical data are represented as means ± S.D. A one-way repeated measures ANOVA followed by Tukey–Kramer multiple comparison test were used to compare the SWVs among three time points in the patients. Factors considered potential confounders were age, sex, and body mass index [13]. Factors considered potential effect modifiers were surgical procedure and use of inhalational or intravenous anesthesia. We also applied multiple linear regression analysis with the threshold *p*-value set to 0.2 to potential factors: age, sex, body mass index, surgical procedure, use of inhalational or intravenous anesthesia. Significant factors were evaluated in subsequent single linear regression models to verify the hypothesis and to perform sensitivity analysis using the other factor (see the Results section). Formulae to correct for the effects of confounders were built on the results of linear regression analyses (see the Results section). All analyses were performed using statistical software (JMP Pro Version 15 for Windows, SAS Institute Inc., Cary, NC, USA). *P* value was considered statistically significant at < 0.05. The theoretical sample size needed for this study was designed to have a power of 1-β: > 0.9 to detect a difference in one-way ANOVA based upon the effect of a 25% change in a standard deviation (0.5 m/s) of SWVs (α < 0.05). Consequently, the sample size was calculated to be 207 measurements (3 groups / 207 measurements = 69 patients).

## Results

### Opioid-induced rigidity increased the abdominal muscle tone and complete muscle relaxation decreased the tone

**Fig. 1:**
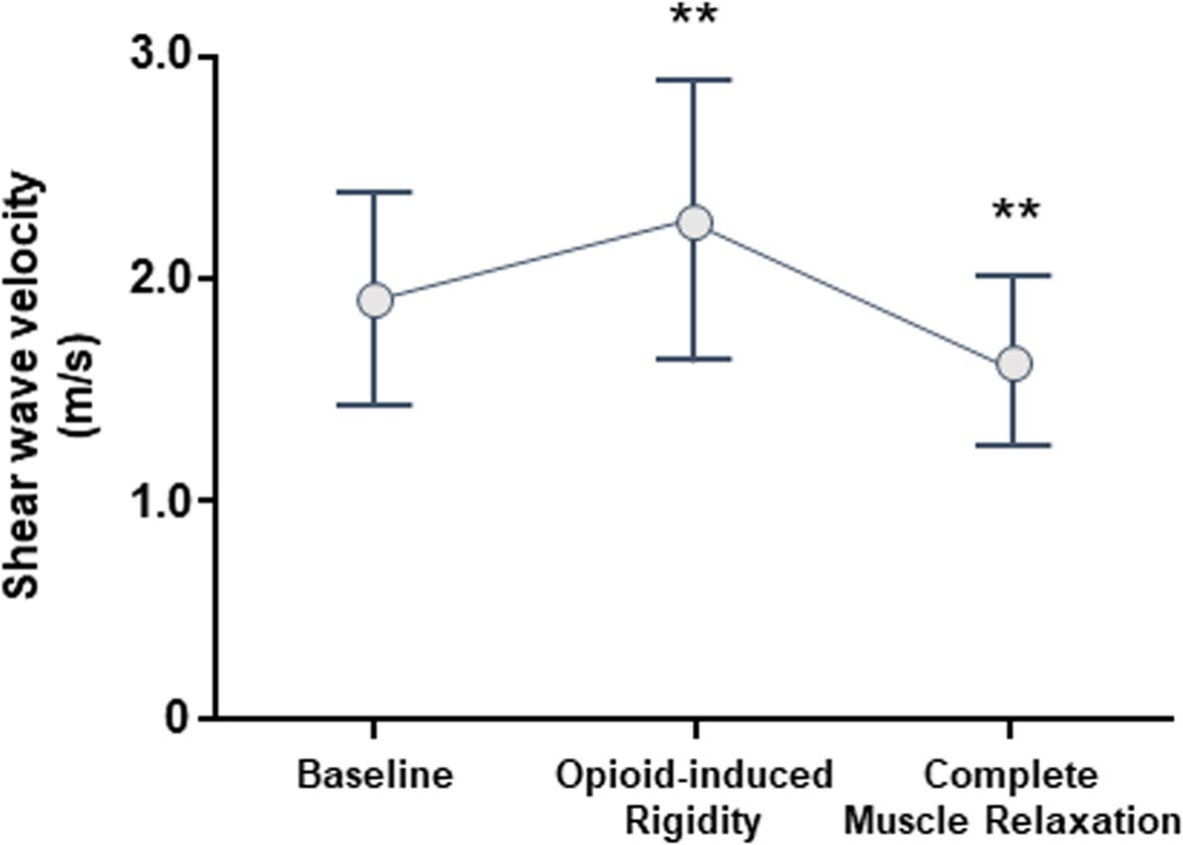
Changes in shear wave velocity during anesthetic induction. The gray circles represent means and the bars represent standard deviations. At the opioid-induce rigidity, the velocity was faster than that at the baseline. At the complete muscle relaxation, the velocity was slower than that at the baseline. **p < 0.01 versus baseline condition

Seventy-five patients were included in the study. Table 1 summarized the demographics of the patients. Figure 1 showed the chronological changes in the SWVs. At the baseline, the SWV was 1.91 ± 0.51 m/s. At the opioid-induce rigidity, the SWV was 2.24 ± 0.65 m/s, and it was faster than that at the baseline (*p* = 0.0005). At the complete muscle relaxation, the SWV was 1.61 ± 0.38 m/s, and it was slower than that at the baseline (*p* = 0.0014).

**Table 1:** Characteristics of the patients

	Data
Total number	75
Age (years)	60 ± 16
Sex	
Male	29 (39%)
Female	46 (61%)
Body mass index	24 ± 4
ASA-PS	
1	7 (9%)
2	50 (67%)
3	18 (24%)
Type of surgery	
Abdominal	57 (76%)
Non-abdominal	18 (24%)
Anesthesia	
Inhalational	32 (43%)
Intravenous	43 (57%)

Data was presented in mean ± standard deviation or numbers (percentage)

### Significant factors affecting the muscle tone were sex and body mass index

To estimate the effects of the coefficients on the SWV and to interrupt selection bias, we used three multiple regression models in each time point (Table 2). Female sex was independent factors in decreasing the SWVs in the both opioid-induced rigidity and complete muscle relaxation. In addition, higher body mass index was independent factors in decreasing the SWVs in the baseline, opioid-induced rigidity and complete muscle relaxation.

**Table 2:** Multivariate models for muscle tone value in anesthetic induction

	Baseline			Opioid-induced rigidity			Complete muscle relaxation		
	Estimate	95%CI	*p* value	Estimate	95%CI	*p* value	Estimate	95%CI	*p* value
Intercept	3.16	2.36 – 3.96	< 0.0001	3.76	2.76 – 4.75	< 0.0001	2.07	1.46 – 2.68	< 0.0001
Age (years)	-0.0009	-0.10 – 0.01	0.84	-0.005	-0.02 – 0.01	0.41	-0.0007	-0.01 – 0.01	0.83
Female	-0.05	-0.18 – 0.07	0.40	-0.21	-0.37 – 0.05	0.01	-0.10	-0.20 – -0.05	0.04
BMI (kg/m^2^)	-0.05	-0.08 – -0.03	0.0002	-0.05	-0.08 – -0.02	0.002	-0.02	-0.04 – 0.00	0.04
Abdominal surgery	-0.07	-0.21 – 0.07	0.31	0.01	-0.16 – 0.18	0.90	0.006	-0.10 – 0.11	0.91
Inhalational anesthesia	0.04	-0.10 – 0.18	0.59	-0.09	-0.26 – 0.08	0.30	0.05	-0.06 – 0.16	0.35

*BMI* body mass index, *95%CI* 95% confidence interval

### The adjustment for subcutaneous fat thickness reduces individual differences in shear wave velocity

**Fig. 2:**
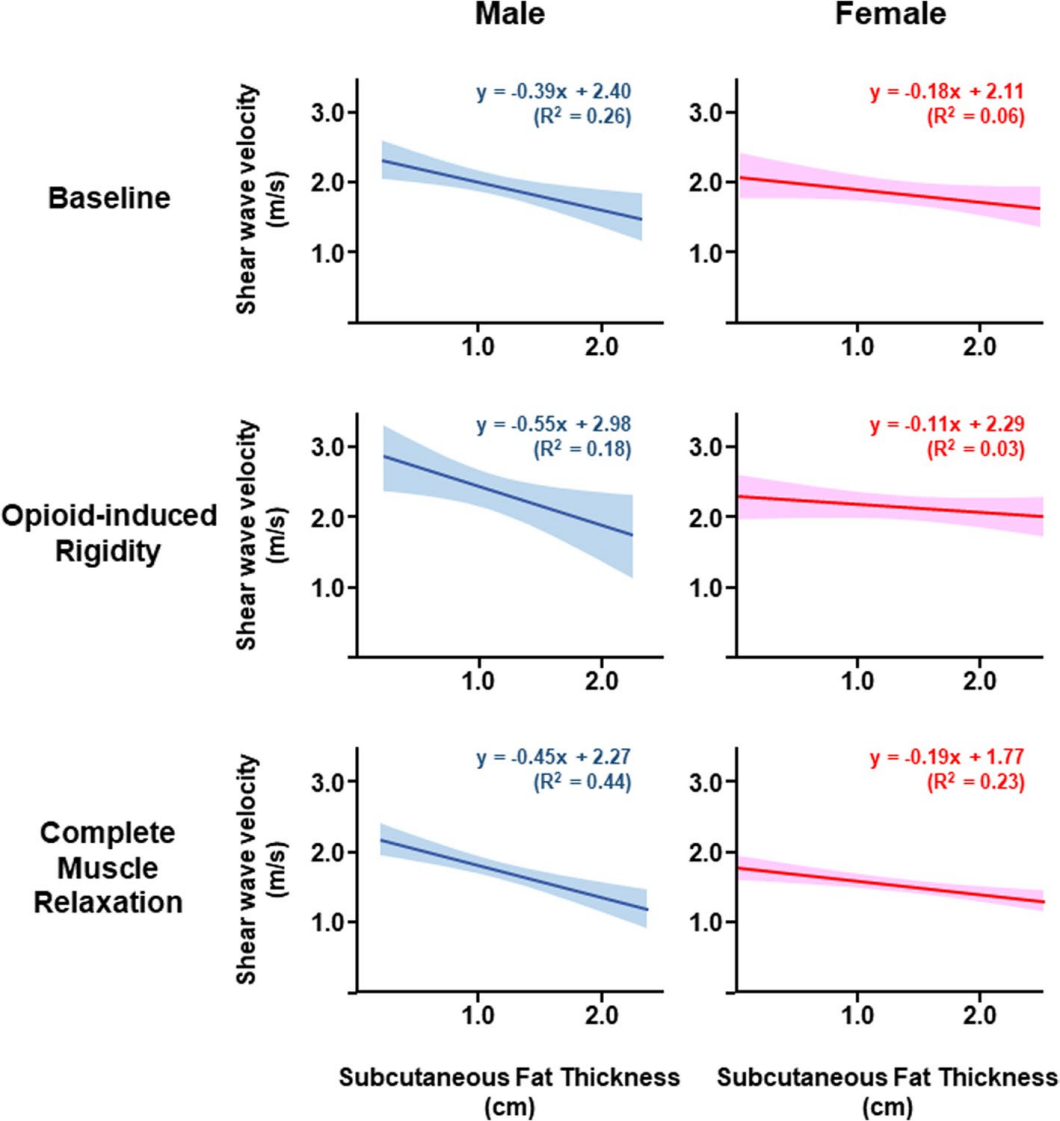
Linear regression models between the subcutaneous fat thickness and shear wave velocity. Six model equations were computed for each of the three situations, for males and females. In the model equation, x is a subcutaneous fat thickness variable, and y is a shear wave velocity variable. Regions around the model line are 95% confidence intervals. R^2^: coefficient of determination

As described above, we found female sex and high body mass index as the significant factors that reduce the SWVs, so we examined whether subcutaneous fat thickness was an alternative potential factor. we constructed a single linear regression model between subcutaneous fat thickness and the SWVs in each sex subpopulation. Figure 2 showed the six models in three-time points. The calculated three y-intercepts in the female were lower than those in the male. The calculated three slopes (i.e., reduction effect of increasing thickness on the SWVs) in the male were higher than those in the female. In the male patients, the reduction effect of increasing thickness on the SWVs was similar among baseline, opioid-induced rigidity, and complete muscle relaxation. In the female patients, the reduction effect was also similar.

In our model of the complete muscle relaxation, if we standardize on being male, having a subcutaneous fat thickness of 1.0 cm, which is average in adult male Japanese [13], the predicted SWV was approximately 1.8 m/s (-0.45 × 1.0 + 2.27). Consequently, we devised the following correction equations so that the SWV approaches 1.8 m/s, canceling the effects of being female and the subcutaneous fat thickness.

$$\begin{array}{ll}Male\ adjusted\ shear\ wave\ velocity\ (m/s) \\ = Measured\ shear\ wave\ velocity\ (m/s)-0.45\times [1-subcutaneous\ fat\ thickness(cm)]\end{array}$$

$$\begin{array}{ll}Female\ adjusted\ shear\ wave\ velocity\ (m/s)\\ = Measured\ shear\ wave\ velocity\ (m/s)-0.19\times \left[1-subcutaneous\ fat\ thickness\left(cm\right)\right]+0.19\end{array}$$

**Fig. 3:**
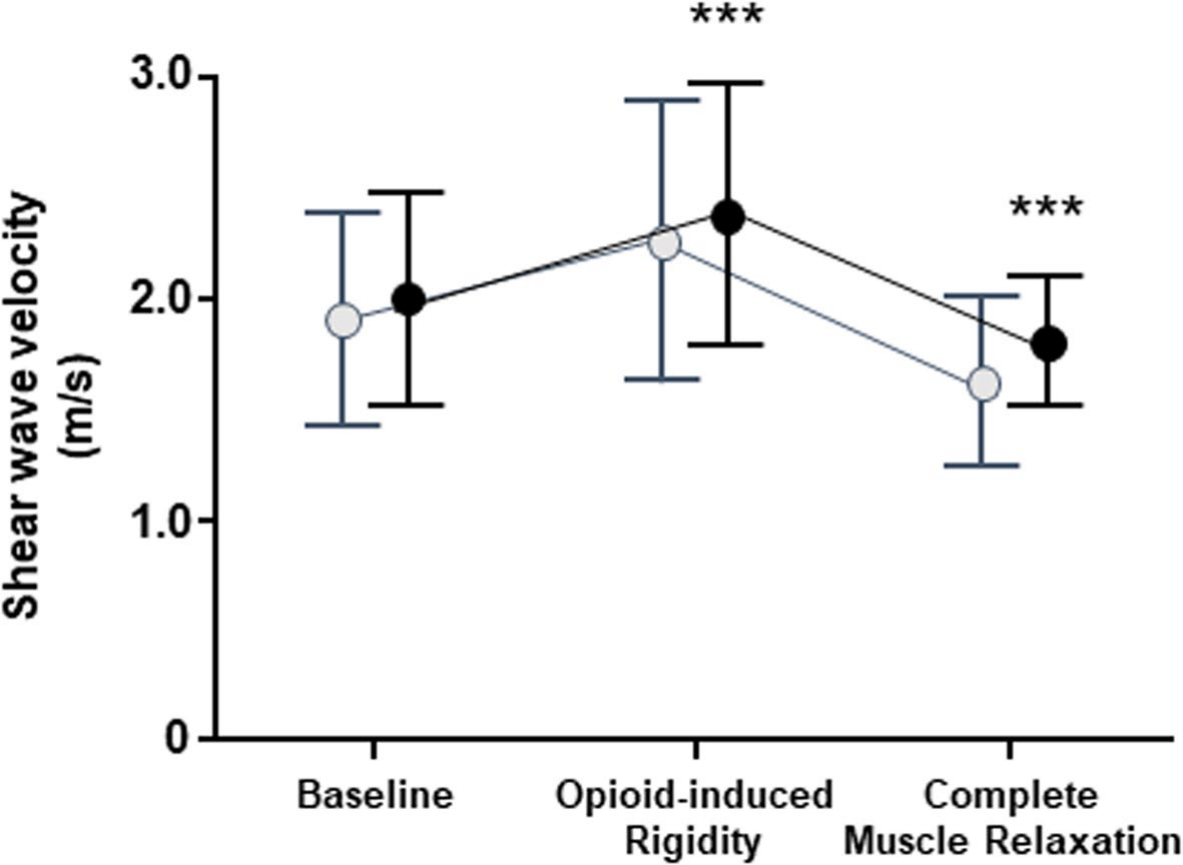
Changes in shear wave velocity after the adjustment. The black circles represent means and the bars represent standard deviations. At the opioid-induce rigidity, the velocity was faster than that at the baseline. At the complete muscle relaxation, the velocity was slower than that at the baseline. Also shown were changes in velocity before adjustment (gray circles and bars, same as Fig. 1). ***p < 0.001 versus baseline condition

Figure 3 showed the chronological changes in the SWVs adjusted for subcutaneous fat thickness. At the baseline, the adjusted SWV was 2.08 ± 0.48 m/s. At the opioid-induced rigidity, the SWV was 2.41 ± 0.60 m/s, and it was faster than that at the baseline (*p* = 0.0001). At the complete muscle relaxation, the SWV was 1.79 ± 0.30 m/s, and it was slower than that at the baseline (*p* = 0.0006).

## Discussion

To develop the universal scale for quantitative assessment of the muscle tone, we first measured and calculated the SWV in three situations. Using the multiple regression model constructed in the current study, we found that female sex and obesity significantly decrease the SWV. In addition, we finally constructed new single regression model and devised a unique adjustment method to improve the measurement accuracy of the SWVs.

The results shown in Fig. 1 indicated the muscle tone levels in the three conditions, which are keystones for the universal scale or quantitative assessment of muscle tone. At baseline condition, the SWV was 1.9 m/s. We assume that it reflects the level of muscle tone at rest, in the patient. Given that the patient was in supine position during measurements, muscle contraction for standing retention and gravity effects are considered negligible [14]. At opioid-induced rigidity conditions, the SWV was 2.2 m/s. However, interpreting this observation is difficult. An earlier report suggested that opioid-induced rigidity was equivalent to muscle stiffness with Parkinsonism [15]. Ding et al*.* reported that the shear wave velocity in the biceps brachii muscle of patients with Parkinson’s disease was 3.7 m/s [16]. This velocity is relatively faster than our results, and so the interpretation may be controversial. We presume that the measured SWV at opioid-induced rigidity condition is the same as muscle tone when one keeps the abdominal muscles tight.

As shown in Table 2, significant factors affecting the muscle tone were sex and body mass index. These two factors were used in the multiple regression model, but may not be independent of each other. Women have more body fat mass than men [17], and obesity is a state of excessive body fat accumulation [18]. Body fat mass may be the potential factor to decrease SWV. Body fat mass is classified into visceral fat and subcutaneous fat [19]. CT scan, MRI, and dual-energy X-ray absorptiometry quantify the visceral fat [19], while skinholds by using a caliper or ultrasound can measure the subcutaneous fat easily [19, 20]. We hypothesized that subcutaneous fat thickness would be a significant factor to reduce SWV based on the interpretation of multiple regression models (Table 2). We referred to the stored ultrasound images in which velocity was measured, and we retrospectively measured subcutaneous fat thickness. As shown in Fig. 2, in the both male and female patients, simple linear regression was fitted between subcutaneous fat thickness and the SWV. Next, using the regression model in the complete muscle relaxation conditions of the male and female, we adjusted the measured SWVs. The results shown in Fig. 3 indicated that the SWV was 1.8 m/s at reference (i.e., zero point), and that the SWV was 2.0 m/s at rest (~ 10% increase from the reference). In the opioid-induced rigidity condition, the SWV was 2.4 m/s (~ 30% increase from the reference). In all conditions, the individual differences were smaller than those before adjustment (Fig. 3), and the SWVs after the adjustment were thought to be useful at least as controls to assess the abdominal muscle tone.

One of the most important components of the proposed scale is the zero point. The adjusted SWV under the complete muscle relaxation condition, that is approximately 1.8 m/s, can be considered as a reference point. So far, to the best of our knowledge, such a reference velocity has not been reported in the past. We therefore compare shear wave velocity to those in softer organ than fully relaxed muscle. Barr et al*.* reported that the velocity in the liver was approximately 1.5 m/s [21, 22]. The value of 1.8 m/s may be reasonable reference point on the scale for assessment of muscle tone.

Our proposed adjustment for subcutaneous fat thickness is practical because it can be measured simultaneously with ultrasound manipulation when measuring velocity, and it is reasonable to assume that, for clinical use of SWV, the thicker the subcutaneous fat, the more likely it is that the ultrasound waves emitted by the echo probe will be attenuated by the subcutaneous fat [23]. It is a plausible explanation that thicker subcutaneous fat reduces SWV. Nevertheless, this adjustment may not be appropriate for other parts of the body, such as the neck, shoulders, waist, and lower limbs, where muscle stiffness often happens in clinical practice [10], because the subcutaneous fat thickness in each site varies even in the same individual [13]. Further investigation at the other sites is needed to generalize our proposed scale. If subcutaneous fat thickness-adjusted scales at the other sites are similar to that at the abdominal muscle, its scale may be universal for assessment of the muscle tone in medicine. For example, the adjusted-SWV values can be used to quantify the severity of conditions such as neck stiffness, frozen shoulder, or back stiffness associated with lower back pain, which are prevalent among middle-aged individuals. Measuring the adjusted-SWV values is also beneficial for identifying muscle weakness, such as ICU-acquired muscle weakness. This would be valuable in clinical practices in orthopaedics, rehabilitation, neurology, intensive care, and pain clinics. Moreover, based on the results of this study, SWV and its scale could potentially serve as an alternative to evaluating muscle relaxation during general anaesthesia, instead of train-of-four monitoring. If the surgical site is other than the abdomen and echo probe sterilization can be ensured, muscle relaxation can be evaluated using methods other than train-of-four monitoring, even in abdominal surgeries.

Moreover, at least based on the results of this study, SWV and its scale are possible to serve as an alternative to the evaluation of muscle relaxation using train-of-four monitoring during general anesthesia. If the surgical site is anywhere other than the abdomen and if echo probe sterilization can be ensured even in the abdomen, muscle relaxation can be evaluated using methods other than train-of-four monitoring.

One of the limitations of this study is the variety of anesthesia methods used during anesthesia induction. Inhaled anesthetics, (i.e., sevoflurane and desflurane), potentiate muscle relaxation and are weak muscle relaxants on their own [24]. Although this patient selection bias influences the measurement of the SWV, our regression models showed no statistically significant effect of inhalation anesthetic (Table 2). We guess that the effect of inhalation anesthetic may be small.

The other limitation of this study is that all measurements were by only one sonography examiner. Ultrasonography depends on the operator’s skill and experience and is not reproducible [25]. In the current study, we did not use the other methods in the measurement of the shear wave velocity and subcutaneous fat thickness except for ultrasound sonography. Also, when the examiner measured the subcutaneous fat thickness, he did not estimate the degree of fat infiltration into the abdominal muscle. Indeed, neuromuscular disease, disuse muscle atrophy, or sarcopenia induce the fat-rich muscle [26]. The possibility that the degree of fat content in muscle is related to muscle tone was not examined. As described above, these information biases may involve the present results in the study.

In conclusion, the present results suggest that shear wave elastography allows for quantification of the muscle tone in the abdomen and that significant clinical factors for decreasing SWV were female sex and high body mass index. The SWV adjusted for the subcutaneous fat thickness may be scale points of future universal scale for the assessment of muscle tone.

## Data Availability

The datasets used during the current study are available from the corresponding author on reasonable request.

## Abbreviations

ASA-PS: American society of anesthesia physical status classification
CT: Computed tomography
ICU: Intensive care unit
MRI: Magnetic resonance imaging
S.D.: Standard deviation
SWV: Shear wave velocity
